# Identification of PI3K‐AKT Pathway‐Related Genes and Construction of Prognostic Prediction Model for ccRCC

**DOI:** 10.1002/cnr2.70010

**Published:** 2024-09-05

**Authors:** Shaowen Hu, Xiaoli Zhang, Huiru Xin, Mingjie Guo, Yafei Xiao, Zhongwei Chang, Qingyang Luo, Yang Li, Chaoyang Zhu

**Affiliations:** ^1^ Department of Urinary Surgery Huaihe Hospital of Henan University Kaifeng China; ^2^ Department of Thoracic and Cardiovascular Surgery Huaihe Hospital of Henan University Kaifeng China; ^3^ Department of Thoracic and Cardiovascular Surgery The First Affiliated Hospital of Henan University Kaifeng China; ^4^ Gastrointestinal Surgery, Huaihe Hospital of Henan University Kaifeng China

**Keywords:** bioinformatics, clear cell renal cell carcinoma, *EFNA3*, *IL2RG*, *MTCP1*, prognostic model, the PI3K‐Akt signaling pathway

## Abstract

**Background:**

Clear cell renal cell carcinoma (ccRCC), the predominate histological type of renal cell carcinoma (RCC), has been extensively studied, with poor prognosis as the stage increases. Research findings consistently indicated that the PI3K‐Akt pathway is commonly dysregulated across various cancer types, including ccRCC. Targeting the PI3K‐Akt pathway held promise as a potential therapeutic approach for treating ccRCC. Development and validation of PI3K‐Akt pathway‐related genes related biomarkers can enhance healthcare management of patients with ccRCC.

**Purpose:**

This study aimed to identify the key genes in the PI3K‐Akt pathway associated with the diagnosis and prognosis of CCRCC using data mining from the Cancer Genome Atlas (TCGA) and Gene Expression Synthesis (GEO) datasets.

**Methods:**

The purpose of this study is to use bioinformatics methods to screen data sets and clinicopathological characteristics associated with ccRCC patients. The exhibited significantly differential expressed genes (DEGs) associated with the PI3K‐Akt pathway were examined by KEGG. In addition, Kaplan–Meier (KM) analysis used to estimate the survival function of the differential genes by using the UALCAN database and graphPad Prism 9.0. And exploring the association between the expression levels of the selected genes and the survival status and time of patients with ccRCC based on SPSS22.0. Finally, a multigene prognostic model was constructed to assess the prognostic risk of ccRCC patients.

**Results:**

A total of 911 genes with common highly expressed were selected based on the GEO and TCGA databases. According to the KEGG pathway analysis, there were 42 genes enriched in PI3K‐Akt signalling pathway. And seven of highly expressed genes were linked to a poor prognosis in ccRCC. And a multigene prognostic model was established based on IL2RG, EFNA3, and MTCP1 synergistic expression might be utilized to predict the survival of ccRCC patients.

**Conclusions:**

Three PI3K‐Akt pathway‐related genes may be helpful to identify the prognosis and molecular characteristics of ccRCC patients and to improve therapeutic regimens, and these risk characteristics might be further applied in the clinic.

## 1 Introduction

According to the International Agency for Research on Cancer (IARC), there was predicted to be 10 million cancer‐related deaths and 13.3 million new cases of cancer worldwide in 2020, heavily affecting public health worldwide [1, 2]. Approximately 3% of all adult malignancies were RCC, one of the most prevalent malignant tumors of the urinary system, whose incidence was rising annually [1]. Clear cell renal cell carcinoma (ccRCC), as the most prevalent pathologic subtype of RCC, was closely related to the pathological stage, with a decreasing trend in recurrence‐free survival from stage I to stage IV [3, 4]. Early surgical treatment is considered the primary treatment for patients with ccRCC [5]. However, approximately 40% of surgical treated patients still develop distant metastasis [4].

In addition, most patients with early renal clear cell carcinoma often showed no notable clinical symptoms, and about 25%–30% of individuals with early RCC had metastases identified at the date of initial diagnosis [6]. For patients with metastatic ccRCC (mccRCC), targeted therapies, immunotherapy, cytoreductive nephrectomy even combined treatment were the primary treatment approaches [7, 8, 9]. Some therapies can increase the chances of survival for patients with renal clear cell cancer. Due to individual differences, lack of reliable prognostic markers and drug resistance, less patients benefit from treatment and more adverse reactions, leading to unsatisfactory treatment results. Therefore, the management of individuals with mccRCC remained a significantly scientific challenge. It was crucial to seek accurate predictive biomarkers to enhance the prospects of patients with ccRCC. Although many biomarkers had been confirmed to be associated with ccRCC survival, no tissue or blood biomarkers had been included in routine clinical practice. However, the clinical transformation of ccRCC‐related diagnostic and prognostic genes continued to expand rapidly. The liquid biopsy (microRNA, exosomes and circulating tumor cells) also showed significant dysregulation in ccRCC, showing great potential in its diagnosis and prognosis and nuclear medicine provided new insights into the possibility of distinguishing benign and malignant lesions and partly distinguished subtypes of RCC [10, 11]. To enhance the care of patients with kidney cancer, these biomarkers still needed to be further explored and confirmed immediately [12].

With the continuous progress of tumor research, many signaling pathways closely related to tumorigenesis and development were constantly being discovered. One of the most prevalent signaling pathways was the phosphoinositide 3‐kinase (PI3K)‐Akt pathway [13]. Numerous investigations revealed that the PI3K‐Akt pathway was abnormally activated in cancer and that it was essential for several biological and cellular processes, including cell proliferation, growth, invasion, migration, and angiogenesis [14, 15]. Under natural circumstances, this pathway was triggered by insulin, growth factors and cytokines and regulated important metabolic processes such as the metabolism of glucose, the synthesis of macromolecules, and the preservation of redox balance to support the growth and metabolism of individual cells as well as the metabolic homeostasis of the entire body. In cancer cells, abnormal activation of PI3K‐Akt pathway increased the activity of metabolic enzymes and nutrition transporters to support process cell metabolism. The PI3K‐Akt pathway was abnormally activated in cancer cells, which increased the expression of metabolic enzymes and nutrient transporters to support process cell metabolism and meet the anabolic needs of cells with abnormal growth [16]. The PI3K‐Akt pathway has been abnormally activated in several malignancies, including ccRCC, gastric tumors, lung tumors, and carcinoma of the prostate [17, 18, 19, 20]. Considering the frequency and significance of the PI3K‐Akt signaling pathway's involvement in kidney cancer, relevant genes in this pathway identified were screened as having diagnostic and prognostic significance in relation to ccRCC for inclusion in the follow‐up study.

In this article, we used bioinformatic methods to take ccRCC patients as the research object in GEO and TCGA database to screen the data set and clinicopathological characteristics related to ccRCC patients, and to screen the genes with diagnosis and prognosis related to ccRCC.

Additionally, we used KEGG to screen for genes associated with the PI3K‐Akt pathway and chose differential genes whose area under curve (AUC) were greater than 0.85, and verified their prognostic significance using UALCAN database, and constructed a multigene prognostic model associated with ccRCC. This study's advancement will enable us to comprehend the mechanisms of ccRCC better, improve the prediction ccRCC better and guide the individualized treatment better.

## 2 Data Collection and Research Methods

### 2.1 Data Source and Collection

The mRNA expression profiles of 534 ccRCC tissues and 72 adjacent normal tissues including age, sex, TNM stage, tumor stage, tumor grade, survival status, and time, were obtained using the TCGA data portal. (https://portal.gdc.cancer.gov/repository/). To verify the accuracy of gene expression, we also conducted a thorough search within the GEO database (https://www.ncbi.nlm.nih.gov/geo/), and found the GSE53757 and GSE66270 datasets in the ccRCC gene expression dataset, respectively. GSE53757 included 72 ccRCC tissues and 72 matching normal kidney tissues, and GSE66270 included 14 ccRCC tissues and 14 matching normal kidney tissues.

### 2.2 Inclusion and Exclusion Criteria

Inclusion: (1) Patients with by ccRCC confirmed by pathological examination and age ≥ 18 years. (2) Patients with complete gene expression data, survival outcomes, and overall survival.

Exclusion: (1) Patients with age < 18 years. (2) Patients with incomplete gene expression data, survival outcomes, and overall survival. (3) Patients with non‐ccRCC or combination of other cancers.

### 2.3 Screening of the DEGs in the ccRCC Dataset

Sangerbox was a bioinformatics processing software based on R language, easily to operate, supporting public data downloads, and preprocessing of clinical sample information in databases [21]. In this study, the Sangerbox software was used to process all the genes of the TCGA‐ccRCC, GSE53757, and GSE66270 datasets and set the cutoff standard with |log2FC > 1| and *p* < 0.05 to obtain differentially expressed genes.

### 2.4 Functional Enrichment Analysis of the Highly DEGs

In order to further obtain highly differential expressed genes (DEGs) associated with PI3K‐Akt pathway, Online mapping software Venny 2.1 (https://bioinfogp.cnb.csic.es/tools/venny/index.html) was utilized to obtain common differential genes of high expression among the three datasets with log2FC > 1 and *p* < 0.05. Then KEGG (http://www.kegg.jp) pathway enrichment analysis was carried out on the common highly expressed differential genes using the Database for Annotation, Visualization and Integrated Discovery database (DAVID, http://www.david.niaid.nih.gov) to find out the main pathway for the enrichment of relevant DEGs. And the DEGs with AUC value >0.85and *p* < 0.05 in PI3K‐Akt pathway were selected for the subsequent screening and validation.

### 2.5 Screening and Validating of Highly DEGs in the PI3K‐AKT Pathway

In order to screen and validate the highly DEGs with prognostic significance related to the PI3K‐Akt pathway, KM survival curves of significant genes in the PI3K‐Akt pathway was downloaded from the KM survival analysis in the UALCAN online cancer analysis platform (http://ualcan.path.uab.edu) to validate the involved genes [22]. The involved genes screened were considered to be significantly associated with ccRCC prognosis with *p* < 0.05. PubMed (https://www.ncbi.nlm.nih.gov/) was used to retrieve relevant articles on differentially expressed genes in the PI3K‐Akt pathway associated with the diagnosis or prognosis of ccRCC, which not investigated were considered as novel genes.

### 2.6 Analyzing and Verifying the Prognostic Value of the New Genes

To verify the prognostic value of novel genes, Kaplan–Meier (KM) survival analysis of novel genes was performed based on TCGA‐ccRCC dataset and the novel genes' KM survival curves were downloaded. The association between the new gene expression level and the survival status and time of ccRCC patients were investigated using the univariate Cox regression analysis.

### 2.7 Construction of the Multigenic Prognostic Model

To learn about the prognostic impact of co‐expression of multiple novel genes on the prognosis of ccRCC, prognostic index (PI) values were calculated and patients were divided into two groups according to the PI scores. KM survival curves were plotted to clarify the difference in survival status between the different expression groups. And the predictive significance of the polygenic prognostic model in patients with ccRCC will be assessed using the Cox regression analysis.

### 2.8 Statistical Analysis

GraphPad Prism 9.0 was used to conduct the survival analysis, ROC analysis, and survival curves. SPSS 20.0 was used to execute the *χ* ^2^ test as well as the univariate and multivariate Cox regression analysis. *p* < 0.05 was regarded as statistically significant.

## 3 Result

### 3.1 Screening of the Differential Genes

**FIGURE 1:**
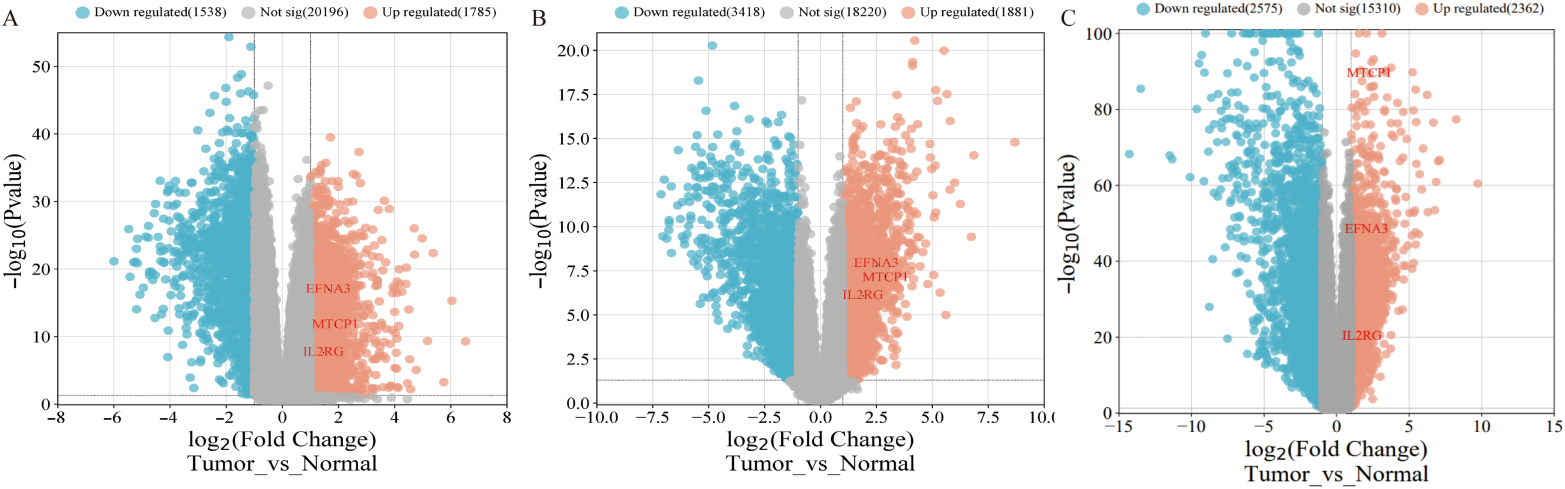
Volcano plots of the expressed genes in the three datasets. Red dots represented up‐regulated genes, blue dots represented down‐regulated genes, and grey dots represented genes not differentially expressed with |log2FC| > 1and *p* < 0.05. (A) The volcano plot drawn based on the dataset GES53757. (B) The volcano plot drawn based on the dataset GES66270. (C) The volcano plot drawn based on the dataset TCGA‐ccRCC.

All gene expression were downloaded from TCGA‐ccRCC dataset. All clinical data of ccRCC were obtained from TCGA‐ccRCC, GSE53757, and GSE66270 datasets, respectively. The TCGA‐ccRCC, GSE53757, and GSE66270 datasets were preliminarily screened using Sangerbox software. And 15 278, 12 971, and 16 901 statistically significantly different genes with *p* < 0.05 were screened, respectively. The volcano visualization of differential genes in each dataset was shown in Figure 1, according to the degree of gene expression.

### 3.2 Functional Enrichment Analysis of Highly DEGs

**FIGURE 2:**
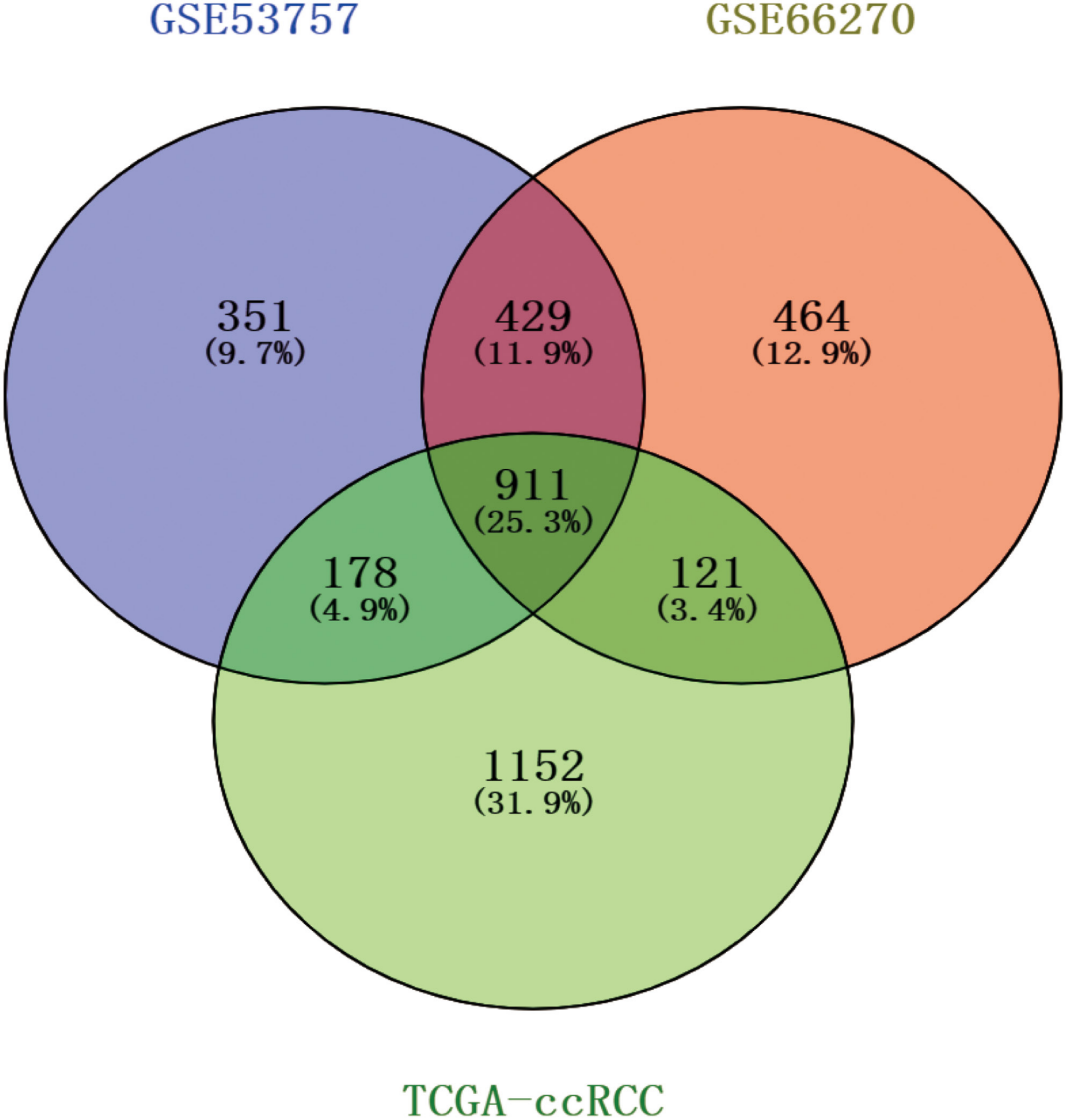
Venn diagram of highly expressed genes in the three datasets. Each color represented highly expressed genes in different datasets, where the crossover regions were genes commonly highly expressed in the different datasets.

**FIGURE 3:**
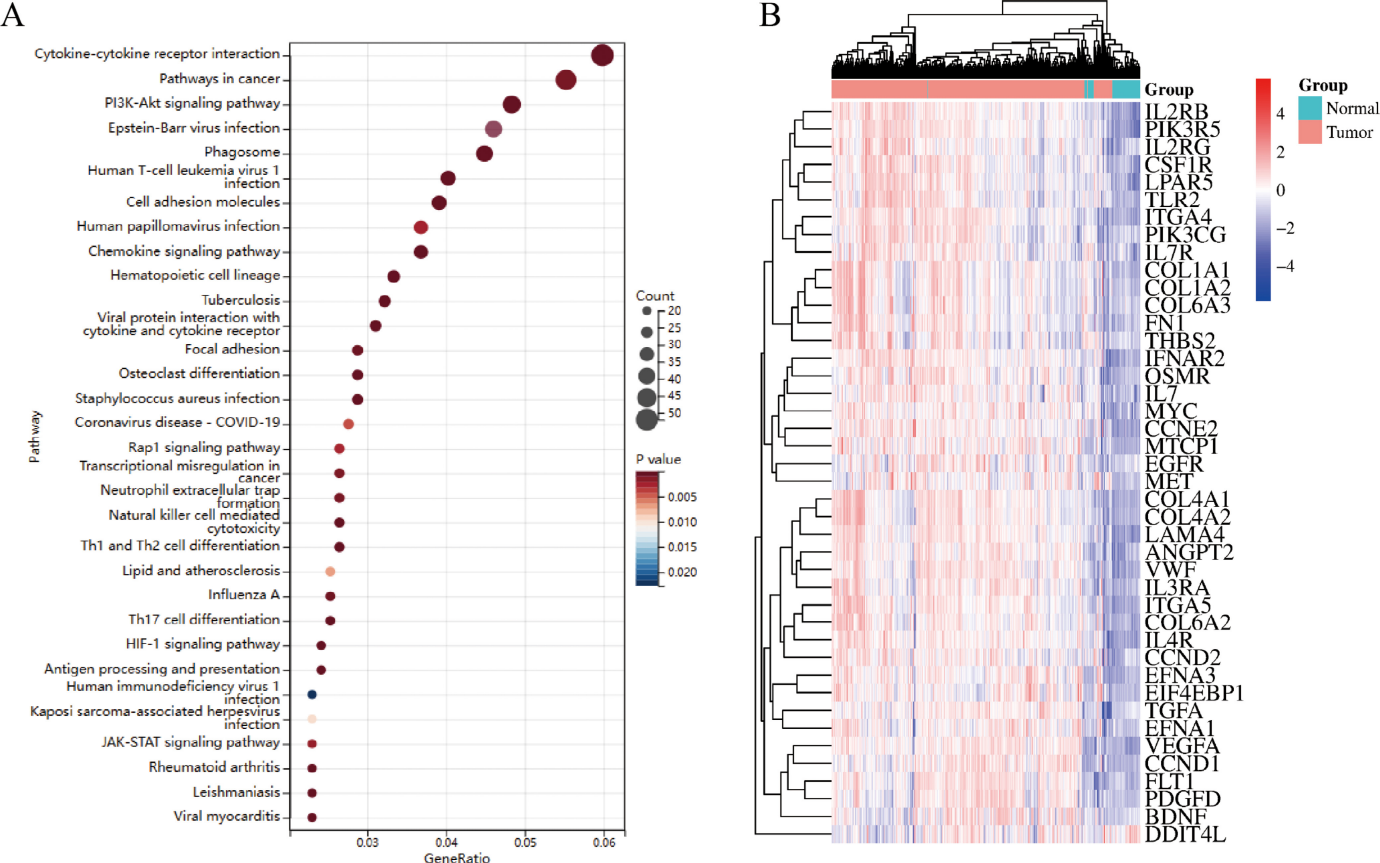
Pathway bubble mapping and expression visualization analysis of differential genes. (A) The signaling pathway enrichment analysis of 911 highly DEGs based on KEGG. The PI3K‐Akt signaling pathway ranked the third based on the gene counts ranking. (B) Analysis of 42 genes involved in the PI3K‐Akt signaling pathway. All of these 42 genes showed highly expressed status in ccRCC.

Secondly, the corresponding Venn diagram was drawn for the differential genes with high expression in the above three datasets. As shown in Figure 2, there were 911 genes with common highly DEGs selected, which indicated that the common highly expression genes selected in the above three datasets could distinguish the kidney cancer disease group and the control group, which had good diagnostic value. The DAVID website was then used to carry out the KEGG enrichment analysis on the 911 genes, with a total enrichment of up to 80 pathways shown in Table S1. The highly DEGs were primarily concentrated in the cytokine–cytokine receptor interaction, pathways in cancer, PI3K‐Akt signaling pathway, Epstein–Barr virus infection, phagosome, human T‐cell leukemia virus 1 infection, cell adhesion molecules, and chemokine signaling pathway, according to the KEGG pathway analysis. Based on the gene counts ranking, we plot the gene counts over 20 involved pathways into the scatter plot shown in Figure 3A. Among these, 42 genes were involved in the PI3K‐Akt pathway, respectively: *CSF1R, FLT1, LAMA4, TGFA, THBS2, IL2RG, EGFR, PIK3CG, PIK3R5, CCND2, CCND1, MYC, PDGFD, EIF4EBP1, IFNAR2, ANGPT2, IL4R, VWF, ITGA4, BDNF, FN1, OSMR, VEGFA, EFNA1, COL1A1, EFNA3, COL1A2, COL4A2, LPAR5, CCNE2, COL4A1, IL7, COL6A2, DDIT4, IL2RB, IL3RA, COL6A3, MTCP1, ITGA5, IL7R, MET, TLR2*. The expression of the above 42 genes were plotted in the TCGA‐ccRCC dataset as shown in Figure 3B based on the heatmap.

### 3.3 Screening and Validation of Highly DEGs in the PI3K‐AKT Pathway

**FIGURE 4:**
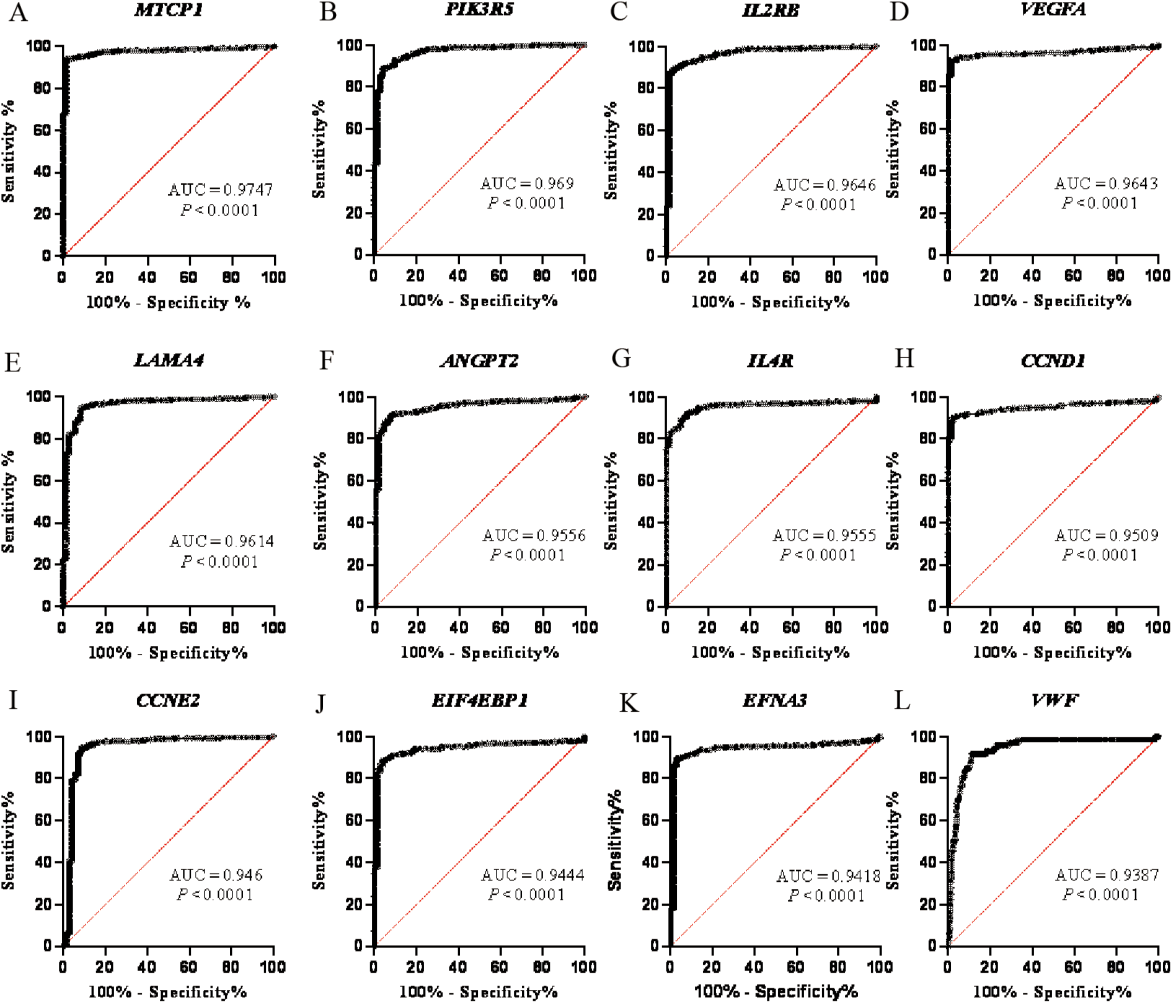
Ranking of the AUC values for the top 12 genes.

**FIGURE 5:**
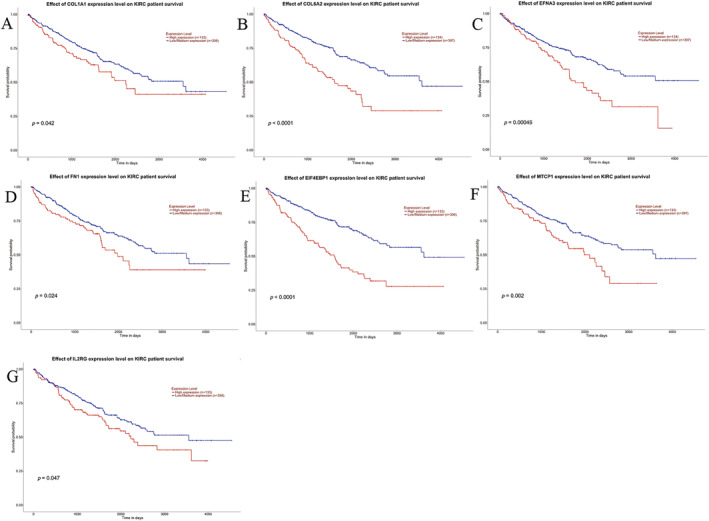
Seven genes clearly associated with poor prognosis in ccRCC.

Based on the TCGA‐ccRCC dataset, these 42 genes were, respectively, analyzed for differential gene expression in sequence, and the ROC curves of the 42 genes were plotted as shown in Figure S1. The ROC curves of the top 12 genes ranked according to their AUC values were listed as shown in Figure 4. Using AUC = 0.85 as the cutoff value, we found that the gene *MTCP1* (AUC = 0.9747) had the largest AUC value, followed by *PIK3R5* (AUC = 0.969), *IL2RB* (AUC = 0.9646), *VEGFA* (AUC = 0.9643), and *LAMA4* (AUC = 0.9614), indicating that these five genes had better diagnostic accuracy for ccRCC and could identify patients with ccRCC from the population easily. Nine of these genes with AUC < 0.85, namely *THBS2* (AUC = 0.6839), *IL7R* (AUC = 0.723), *COL6A3* (AUC = 0.7419), *BDNF* (AUC = 0.7627), *PIK3CG* (AUC = 0.7758), *EGFR* (AUC = 0.7975), *COL1A2* (AUC = 0.8059), *CCND2* (AUC = 0.8373), and *OSMR* (AUC = 0.8495), indicated that these nine genes had poor diagnostic accuracy and might be likely to lead to misdiagnosis of ccRCC. After removing these 9 genes, the remaining 33 genes were included for sequential prognostic analysis using the UALCAN website and the corresponding KM survival curves were downloaded shown in Figure S2. We found that 12 genes were statistically strongly correlated with the ccRCC patients' survival status with *p* < 0.05, namely *CCND1, COL1A1, COL6A2, EFNA3, EIF4EBP1, FLT1, FN1, IL2RG, MTCP1, PDGFD, TGFA*, and *VWF*. Seven of these genes were associated with poor prognosis in ccRCC when highly expressed, namely *MTCP1* (*p* = 0.002), *EIF4EBP1* (*p* < 0.0001), *FN1* (*p* = 0.024), *COL1A1* (*p* = 0.042), *EFNA3* (*p* = 0.00046), *COL6A2* (*p* < 0.0001), and *IL2RG* (*p* = 0.047) shown in Figure 5. This suggested that these seven genes were able to estimate the survival rate of individuals with kidney cancer with good prognostic significance.

On October 1, 2023, PubMed was used to search the literature for the above seven genes with prognostic significance for ccRCC patients in relation to ccRCC using the search terms “clear cell renal carcinoma,” “renal clear cell carcinoma,” “ccRCC,” and “KIRC,” which showed that *MTCP1*, *EFNA3*, and *IL2RG* had not yet been investigated and were considered to be novel genes related to diagnosis and prognosis of ccRCC patients.

### 3.4 Prognostic Value Analysis and Validation of New Genes

**FIGURE 6:**
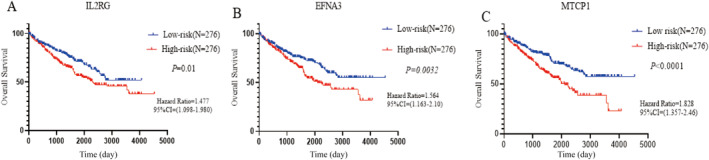
Prognostic analysis of novel genes based on the TCGA‐ccRCC dataset.

The expression levels of genes *MTCP1*, *EFNA3*, and *IL2RG* and related clinicopathologic features of ccRCC patients were downloaded according to the TCGA‐ccRCC dataset. Total 534 patients with ccRCC data were included after removing no gene expression values and control group data. We categorized the samples into the high‐expression (*N* = 267) and low‐expression groups (*N* = 267) according to the median mRNA expression levels of *MTCP1*, *EFNA3*, and *IL2RG* genes. To ascertain the importance of new genes expression for prognosis in patients with ccRCC, we included patient survival status and gene expression levels in KM analysis and the univariate Cox regression analysis. KM analysis, as shown in the Figure 6, demonstrated that all of the novel genes' expression levels were significantly correlated with the survival of patients with ccRCC, and hazard ratios (HR) for the gene expression levels of *MTCP1*, E*FNA3*, and *IL2RG* were 1.828, 1.564, and 1.477, respectively, which indicated that compared to patients in the group with the low expression level of the new genes, the patients in the high expression group, which corresponded to the high expression level of the new genes, had a shorter survival time. The univariate Cox regression analysis shown in Table 1, *IL2RG* expression level (HR = 1.484, 95%CI = 1.097–2.009, *p* = 0.011), *EFNA3* expression level (HR = 1.569, 95%CI = 1.116–2.122, *p* = 0.003), and *MTCP1* expression level (HR = 1.847, 95%CI = 1.361–2.506, *p* < 0.001) showed a strong correlation with the patients' overall survival time in ccRCC (*p* < 0.05).

**TABLE 1:** The univariate Cox regression analysis for *IL2RG*, *EFNA3*, and *MTCP1*.

Gene	*B*	Univariate Cox regression analysis		
		HR	95% CI	*p*
*IL2RG*	0.395	1.484	1.097–2.009	0.011
*EFNA3*	0.45	1.569	1.116–2.122	0.003
*MTCP1*	0.614	1.847	1.361–2.506	<0.001

### 3.5 Construction of the Multigenic Prognostic Model

**FIGURE 7:**
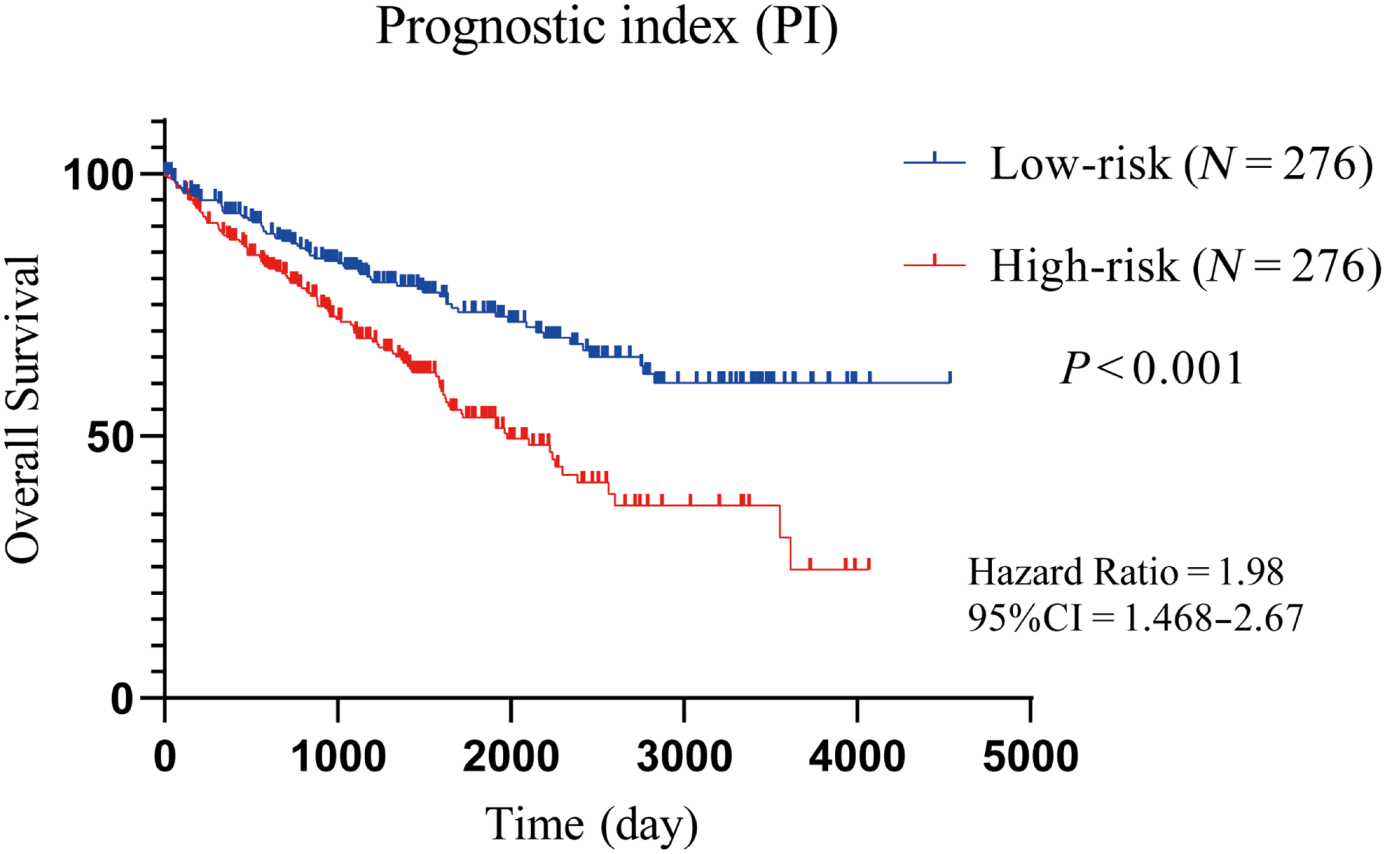
Prognostic analysis of novel genes based on the prognostic index in ccRCC.

To further analyze the effect of *IL2RG*, *EFNA3*, and *MTCP1* co‐expression on the survival time of ccRCC patients, we first carried out the establishment of a multigene prognostic model, according to the PI formula, PI (*n*) = *β*′1X′*n*1 + *β*′2X′*n*2 + *β*′3 *X*′*n*3, where the “*β*” value was the regression coefficient *B* value of the independent variable (mRNA) originated from the univariate Cox analysis of the three genes, and “*X*” denoted the expression of mRNA. The formula for the prognostic score was PI = 0.395 × X (*IL2RG*) + 0.45 × X (*EFNA3*) + 0.614 × X (*MTCP1*). Using the median value of the PI as the cut‐off point, the patients with ccRCC were divided into two groups: those with high expression and those with low expression. 267 patients with a score ≤ 9.935897 were included in the low‐risk group, and 267 patients with a score > 9.935897 were included in the high‐risk group. The KM survival curves were then plotted with GraphPad software according to the grouping, and the findings demonstrated that patients in the low‐risk group had a greater overall survival rate than those in the high‐risk group (*p* < 0.001). To further determine whether the prognostic model could be an independent prognostic factor for ccRCC patients, we performed *χ*² test, univariate, and multivariate Cox regression analysis. The results of the *χ*² test were shown in Table 2, the PI was statistically different from the TNM stage of ccRCC patients in terms of tumor size (*p* < 0.001), presence of distant metastasis (*p* = 0.005), tumor stage (*p* < 0.001), tumor grade (*p* < 0.001) and survival status (*p* < 0.001). However, there was no significant statistical difference with age and lymph node metastasis. After that, the above‐mentioned clinical characteristics were submitted to the univariate and multivariate Cox regression analysis as shown in Table 3. The univariate Cox regression analysis's findings revealed that Age (HR = 1.818, 95%CI = 1.329–2.488, *p* < 0.001), T(HR = 3.167, 95%CI = 2.341–4.284, *p* < 0.001), N (HR = 3.392, 95%CI = 1.801–6.389, *p* < 0.001), M(HR = 4.351, 95%CI = 3.190–5.935, *p* < 0.001), stage (HR = 3.854, 95%CI = 2.811–5.284, *p* < 0.001), Ggade (HR = 2.615, 95%CI = 1.862–3.673, *P*<0.001), and PI (HR = 2.116, 95%CI = 1.551–2.887, *p* < 0.001) were closely related to the OS. The multivariate Cox regression analysis's findings revealed that T(HR = 1.865, 95%CI = 1.182–2.943, *p* = 0.007), M(HR = 2.806, 95%CI = 1.772–4.445, *p* < 0.001), Grade (HR = 1.638, 95%CI = 1.007–2.663, *p* < 0.001), PI (HR = 1.653, 95%CI = 1.075–2.542, *p* = 0.022)were independent predictors of the patients' prognosis with ccRCC. Hazard ratio of T stage was 2.116, suggesting that the prognosis of patients with T3‐4 was worse than that with T1‐2. Hazard ratio of M stage was 2.806, claiming that individuals who had distant metastases had a poorer prognosis than those who did not. The hazard ratio of Grade was 1.638, suggesting that patients with GIII‐IV stage had a worse prognosis than patients with GI‐II stage. The hazard ratio of PI was 1.653, suggesting that the prognosis for patients in the group with higher risk was worse than for those in the group with lower risk. Finally, again using the KM analysis, where ccRCC was divided into different groups according to the median PI values, we found that overall survival in the group with high‐risk was worse than that in the group with low‐risk, as shown in Figure 7. The analysis's findings indicated that the prognostic gene model may be utilized independently of other prognostic markers to assess the survival of patients with ccRCC.

**TABLE 2:** The *χ*² test of prognostic index and clinicopathological characteristics.

Clinical characteristics	Groups	Prognostic index (PI)		*χ*² value	*p*
		High‐expression (*n* = 267)	Low‐expression (*n* = 267)		
Age	<60 ≥60	117 150	129 138	1.085	0.340
Gender	Female Male	83 184	105 162	3.973	0.057
T	T1‐2 T3‐4	147 120	196 71	19.571	<0.001
N	N0 N1‐3 Null	120 9 138	120 7 140	0.797	0.234
M	M0 M1 Null	199 51 17	223 28 16	8.059	0.005
Stage	I‐II III‐IV Null	134 131 2	191 76 0	24.603	<0.001
Grade	GI‐II GIII‐IV Null	97 168 2	146 115 6	19.777	<0.001
Survival state	Living Dead	155 112	204 63	20.408	<0.001

**TABLE 3:** The univariate and multivariate Cox survival analysis of prognostic index and clinicopathological characteristics.

Clinical characteristics	Univariate Cox regression analysis		Univariate Cox regression analysis	
	HR (95%CI)	*p*	HR (95%CI)	*p*
Age ≥60 versus <60	1.818 (1.329–2.488)	<0.001		
Gender Male versus Female	0.944 (0.694–1.284)	0.714		
T T3‐4 versus TI‐2	3.167 (2.341–4.284)	<0.001	1.865 (1.182–2.943)	0.007
N N1‐3 versus N0	3.392 (1.801–6.389)	<0.001		
M M1 versus M0	4.351 (3.190–5.935)	<0.001	2.806 (1.772–4.445)	<0.001
Stage III‐IV versus I‐II	3.854 (2.811–5.284)	<0.001		
Grade III‐IV versus I‐II	2.615 (1.862–3.673)	<0.001	1.638 (1.007–2.663)	0.047
Prognostic index (PI) High‐risk versus Low‐risk	2.116 (1.551–2.887)	<0.001	1.653 (1.075–2.542)	0.022

## 4 Discussion

Knowing as the most common malignant subtype of kidney cancer, renal clear cell carcinoma was very necessary for the surgical treatment that can be needed [23]. Distant metastases occurred in more than 30% of ccRCC during treatment [24]. Due to its extremely dismal prognosis—a 5‐year survival rate of less than 20%—the mccRCC was regarded as a deadly illness [25]. In addition, mccRCC was resistant to traditional chemotherapy and radiotherapy, so new treatments and individualized treatments were particularly important [26, 27]. Thankfully, big data, biological informatics, and AI have banded together to provide notable progress in translational medicine and our basic understanding of cancer biology. Furthermore, large‐scale genomic, genomic, and proteomic studies in renal cell carcinoma (RCC) demonstrated the molecular basis of the disease, providing new therapeutic prospects, and new therapeutic opportunities for this challenging disease, as well as promoting new research and ideas [28]. Particularly prominent were immunotherapy and targeted therapies, both of which had made significant contributions to the treatment of mccRCC [29]. Currently, the main types of cancer treatment remained surgery, chemotherapy and radiation therapy, and immunotherapy was emerging as an important modality of cancer treatment [30].

For instance, the U.S. Food and Drug Administration (FDA) authorized the first immunotherapy for RCC, which was to inhibit metastatic RCC progression by using high‐dose interleukin‐2 (IL‐2), which has the ability to stimulate CD8 T lymphocytes to promote their activation to enhance tumor killing [31]; On the other hand, immune checkpoint inhibitors (ICIs) such as anti‐programmed cell death 1 (PD‐1), anti‐programmed death ligand 1 (PD‐L1), and cytotoxic T‐lymphocyte‐associated protein 4 (CTLA‐4) antibodies were used to block the progression of metastatic RCC had been successfully developed and used in the clinic [32]. Targeted therapies, also known as “molecularly targeted therapies,” worked by targeting signaling pathways, angiogenesis, or acting as toxin delivery molecules that interfered with specific proteins required for tumor cell growth or spread in the body [33, 34, 35]. For example, sunitinib, sorafenib can target the VEGFA/VEGFR pathway and neoangiogenesis tyrosine kinase inhibitors (TKI) for the treatment of patients with ccRCC [36, 37]. Diclomax and everolimus can be used as inhibitors of mammalian target of rapamycin (mTOR) to prolong progression‐free survival of patients with ccRCC by regulating hypoxia‐inducible factors associated metabolism and cellular proliferation through the PI3K‐Akt pathway [38, 39]. Although immunotherapy, targeted therapies, and their combinations had proven effective, and novel drugs that enhance tumor/immune system interactions and simultaneously targeted multiple cancer‐causing pathways hold great promise, most patients had drug resistance in targeted therapy and immunotherapy, and almost all patients treated with currently approved targeted drugs would eventually develop disease progression [40, 41, 42]. Therefore, the development of new therapeutic targets may be a possible way to potentially further treat ccRCC.

Hyperactivation of the PI3K‐Akt pathway has been identified as a critical element in the development of tumors. And this pathway frequently activated as a cancer driver, targeting more pathway components, and more tumor types compared to other growth factor signaling pathways [28, 43]. The PI3K‐Akt signaling pathway was an important signaling mechanism involved in cell growth, survival and metabolism [44]. The PI3K is activated by a variety of hormones and growth factors and subsequently phosphorylated phosphatidylinositol‐4,5‐bisphosphate (PIP2) to phosphatidylinositol‐3,4,5‐trisphosphate (PIP3).The Akt, as a PI3K downstream target gene, was an essential cell growth regulator, survival and signaling, and PIP3 can activate Akt kinase through phosphorylation to regulate a variety of downstream target genes, for examples, transcription factors (FOXO), cell cycle regulators (p21 and p27) and components of the mTOR pathway, which further regulated different pathological processes such as cell proliferation, migration, and drug resistance [45, 46].

As therapeutic approaches continued to advance, the identification and validation of biomarkers was critical to optimize first‐line selection and treatment sequencing [40]. There were reports in the literature about the significance of biomarkers in the PI3K‐Akt pathway in the diagnosis, treatment, and prognosis of ccRCC. Some important transcriptional genes were involved in the occurrence and development of ccRCC, for example, in the Von Hippel‐Lindau (VHL) gene‐mediated PI3K‐Akt pathway, deletion or mutation of VHL results in up‐regulation of the PI3K pathway to make ccRCC more aggressive [47]. Knockdown of PLAUR effectively induced apoptosis, regulated the cell cycle, inhibited the EMT process, attenuated the activation of the PI3K‐AKT‐mTOR signaling pathway and ultimately inhibited ccRCC progression [48]. The gene IFI16 promoted the transcription and translation of IL6, which subsequently activated the PI3K‐Akt signaling pathway, induced EMT process, and promoted the progression of ccRCC [49]. Some noncoding RNAs also affected ccRCC development by targeting the PI3K‐Akt pathway, for example, miR‐153‐5p depletion remarkably inhibited the proliferation and metastasis of ccRCC via the PI3K‐Akt signaling [50]. Due to the prevalence of the PI3K‐Akt pathway in tumorigenesis and its criticality to the development of ccRCC, the PI3K isoform inhibitors, AKTs, mTORs, and other components of the pathway were being actively pursued for targeted cancer therapies [28]. In order to explore the interrelationship between related genes in the PI3K‐Akt pathway and the diagnosis and prognosis of ccRCC, we first identified 911 common highly differentially expressed genes using TCGA and GEO databases, then screened related genes involved in the PI3K‐Akt pathway using the DAVID database, and further verified the prognostic significance of the related genes using the UALCAN database. After searching for related articles, we finally identified three PI3K‐Akt pathway‐related genes (*MTCP1*, *EFNA3*, and *IL2RG*) and developed a risk model based on the three genes' expression levels to forecast the prognosis of ccRCC. By classifying individuals with ccRCC into high‐risk and low‐risk groups based on the TCGA‐ccRCC dataset with a median risks, we found that there was a statistically significant difference in OS between patients in the high‐risk and low‐risk groups. Finally, we confirmed that the three PI3K‐Akt pathway‐related gene profiles were an independent prognostic indicator for ccRCC patients. However, there were some limitations of this study. (1) Limitations of sample sources: the clinical data and samples used in this study were mainly from public databases (TCGA and GEO), which may have limited the diversity and representativeness of the data. Future studies should consider using multicenter data from different regions and ethnicities to validate our findings and improve the generalizability of the model. (2) Dependence on bioinformatic methods: although bioinformatic methods can effectively screen and analyze genes from large‐scale data, the method relies on existing databases and algorithms, which may be limited by the quality of the data and the tools used for analysis. Therefore, laboratory validation was essential to confirm our bioinformatic findings. (3) Validation of predictive models: although statistically significant, the multigene prognostic models we constructed have only been validated within databases and have not yet been prospectively validated in independent cohorts or clinical trials. Future studies need to validate the model in a broader patient population to ensure its clinical applicability and accuracy. (4) Functional mechanisms of potential biomarkers were not fully resolved: although we identified biomarkers associated with the PI3K‐Akt pathway, their specific biological functions and mechanisms of action in ccRCC still need to be further explored through basic research.

## 5 Conclusion

In this study, we identified three PI3K‐Akt pathway‐related genes (*MTCP1*, *EFNA3*, and *IL2RG*) that were significantly associated with clinical outcomes in ccRCC using the TCGA and GEO datasets, and constructed prognostic gene models based on the three genes that could be used independently of other conventional clinical factors in patients with ccRCC as an independent prognostic factor for evaluating the survival of patients with ccRCC.

## Author Contributions

S.H., X.Z., and H.X. are the co‐first authors of this article. SH contributed all the figures, X.Z. contributed all the tables, H.X. contributed the supplementary material, all three co‐first authors made significant contributions to the manuscript. Corresponding author C.Z. conceived the study and provided theoretical guidance. M.G., Y.X., Z.C., Q.L., and Y.L. added and supplemented the article. All authors approved the latest version and decided to submit the manuscript.

## Ethics Statement

This paper does not involve experiments and does not require ethical approval.

## Consent

All authors warrant that the manuscript is original and not published yet.

## Conflicts of Interest

The authors declare no conflicts of interest.

## Supporting information


**Data S1.** Supporting information.

## Data Availability

The data and materials presented in this study had been included in the article or supplementary materials. For further details, please contact the authors.
