# Supplementary material for: Identification of PI3K‐AKT Pathway‐Related Genes and Construction of Prognostic Prediction Model for ccRCC
Source: Cancer Rep (Hoboken). 2024 Sep 5;7(9):e70010. doi: 10.1002/cnr2.70010 (PMC11375326; doi:10.1002/cnr2.70010)
Supplement: Supplementary file 1 — Data S1. Supporting information. [file CNR2-7-e70010-s001.docx]

**Supplementary materials:**

**Table S1. KEGG pathway enrichment analysis of 911 genes with common highly differential expression based on TCGA and GEO datasets.**

| **Rank** | **Term** | **Count** | **P Value** |
| --- | --- | --- | --- |
| 1 | hsa04060:Cytokine-cytokine receptor interaction | 52 | 1.17E-13 |
| 2 | hsa05200:Pathways in cancer | 48 | 6.15E-04 |
| 3 | hsa04151:PI3K-Akt signaling pathway | 42 | 4.90E-06 |
| 4 | hsa05169:Epstein-Barr virus infection | 40 | 2.51E-12 |
| 5 | hsa04145:Phagosome | 39 | 7.45E-16 |
| 6 | hsa05166:Human T-cell leukemia virus 1 infection | 35 | 3.58E-08 |
| 7 | hsa04514:Cell adhesion molecules | 34 | 1.41E-11 |
| 8 | hsa04062:Chemokine signaling pathway | 32 | 4.22E-08 |
| 9 | hsa05165:Human papillomavirus infection | 32 | 0.002235 |
| 10 | hsa04640:Hematopoietic cell lineage | 29 | 1.94E-13 |
| 11 | hsa05152:Tuberculosis | 28 | 1.44E-06 |
| 12 | hsa04061:Viral protein interaction with cytokine and cytokine receptor | 27 | 1.21E-11 |
| 13 | hsa05150:Staphylococcus aureus infection | 25 | 1.85E-10 |
| 14 | hsa04380:Osteoclast differentiation | 25 | 2.36E-07 |
| 15 | hsa04510:Focal adhesion | 25 | 2.78E-04 |
| 16 | hsa05171:Coronavirus disease - COVID-19 | 24 | 0.004009 |
| 17 | hsa04658:Th1 and Th2 cell differentiation | 23 | 2.66E-09 |
| 18 | hsa04650:Natural killer cell mediated cytotoxicity | 23 | 1.19E-06 |
| 19 | hsa04613:Neutrophil extracellular trap formation | 23 | 7.08E-04 |
| 20 | hsa05202:Transcriptional misregulation in cancer | 23 | 8.16E-04 |
| 21 | hsa04015:Rap1 signaling pathway | 23 | 0.002447 |
| 22 | hsa04659:Th17 cell differentiation | 22 | 2.85E-07 |
| 23 | hsa05164:Influenza A | 22 | 3.96E-04 |
| 24 | hsa05417:Lipid and atherosclerosis | 22 | 0.00685 |
| 25 | hsa04612:Antigen processing and presentation | 21 | 3.86E-09 |
| 26 | hsa04066:HIF-1 signaling pathway | 21 | 1.44E-06 |
| 27 | hsa05416:Viral myocarditis | 20 | 1.81E-10 |
| 28 | hsa05140:Leishmaniasis | 20 | 1.89E-08 |
| 29 | hsa05323:Rheumatoid arthritis | 20 | 4.72E-07 |
| 30 | hsa04630:JAK-STAT signaling pathway | 20 | 0.001728 |
| 31 | hsa05167:Kaposi sarcoma-associated herpesvirus infection | 20 | 0.00957 |
| 32 | hsa05170:Human immunodeficiency virus 1 infection | 20 | 0.022713 |
| 33 | hsa05145:Toxoplasmosis | 19 | 2.84E-05 |
| 34 | hsa04670:Leukocyte transendothelial migration | 19 | 4.63E-05 |
| 35 | hsa04621:NOD-like receptor signaling pathway | 19 | 0.012896 |
| 36 | hsa05205:Proteoglycans in cancer | 19 | 0.03124 |
| 37 | hsa05163:Human cytomegalovirus infection | 19 | 0.066508 |
| 38 | hsa05135:Yersinia infection | 18 | 0.001233 |
| 39 | hsa05162:Measles | 17 | 0.003417 |
| 40 | hsa04218:Cellular senescence | 17 | 0.011028 |
| 41 | hsa05330:Allograft rejection | 16 | 4.46E-10 |
| 42 | hsa05332:Graft-versus-host disease | 16 | 2.32E-09 |
| 43 | hsa04672:Intestinal immune network for IgA production | 16 | 2.60E-08 |
| 44 | hsa04610:Complement and coagulation cascades | 16 | 5.53E-05 |
| 45 | hsa04974:Protein digestion and absorption | 16 | 4.41E-04 |
| 46 | hsa05322:Systemic lupus erythematosus | 16 | 0.007665 |
| 47 | hsa05340:Primary immunodeficiency | 15 | 5.01E-09 |
| 48 | hsa04940:Type I diabetes mellitus | 15 | 3.15E-08 |
| 49 | hsa05320:Autoimmune thyroid disease | 15 | 5.83E-07 |
| 50 | hsa05321:Inflammatory bowel disease | 15 | 8.07E-06 |
| 51 | hsa04660:T cell receptor signaling pathway | 15 | 0.006092 |
| 52 | hsa04611:Platelet activation | 15 | 0.007545 |
| 53 | hsa04110:Cell cycle | 15 | 0.047829 |
| 54 | hsa04512:ECM-receptor interaction | 14 | 0.001015 |
| 55 | hsa05146:Amoebiasis | 14 | 0.003544 |
| 56 | hsa04620:Toll-like receptor signaling pathway | 14 | 0.0058 |
| 57 | hsa04668:TNF signaling pathway | 14 | 0.009081 |
| 58 | hsa05418:Fluid shear stress and atherosclerosis | 14 | 0.040137 |
| 59 | hsa05133:Pertussis | 13 | 7.78E-04 |
| 60 | hsa04662:B cell receptor signaling pathway | 13 | 0.001908 |
| 61 | hsa04933:AGE-RAGE signaling pathway in diabetic complications | 13 | 0.008072 |
| 62 | hsa04064:NF-kappa B signaling pathway | 13 | 0.01092 |
| 63 | hsa05144:Malaria | 12 | 6.16E-05 |
| 64 | hsa04666:Fc gamma R-mediated phagocytosis | 12 | 0.01634 |
| 65 | hsa05142:Chagas disease | 12 | 0.022976 |
| 66 | hsa05235:PD-L1 expression and PD-1 checkpoint pathway in cancer | 11 | 0.02279 |
| 67 | hsa05134:Legionellosis | 10 | 0.003059 |
| 68 | hsa05222:Small cell lung cancer | 10 | 0.06321 |
| 69 | hsa05310:Asthma | 9 | 1.93E-04 |
| 70 | hsa04979:Cholesterol metabolism | 9 | 0.005972 |
| 71 | hsa05221:Acute myeloid leukemia | 9 | 0.028729 |
| 72 | hsa05230:Central carbon metabolism in cancer | 9 | 0.036133 |
| 73 | hsa04115:p53 signaling pathway | 9 | 0.047867 |
| 74 | hsa04623:Cytosolic DNA-sensing pathway | 9 | 0.051147 |
| 75 | hsa05212:Pancreatic cancer | 9 | 0.054568 |
| 76 | hsa04664:Fc epsilon RI signaling pathway | 8 | 0.076559 |
| 77 | hsa00051:Fructose and mannose metabolism | 6 | 0.035752 |
| 78 | hsa05143:African trypanosomiasis | 6 | 0.049073 |
| 79 | hsa05219:Bladder cancer | 6 | 0.070818 |
| 80 | hsa03260:Virion - Human immunodeficiency virus | 3 | 0.026667 |

**Figure S1 ROC curves of the 42 genes in PI3K-Akt pathway based on the TCGA-ccRCC dataset.**


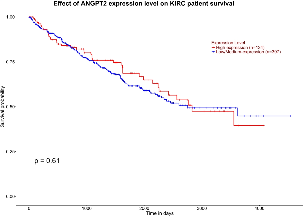

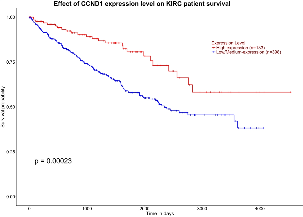

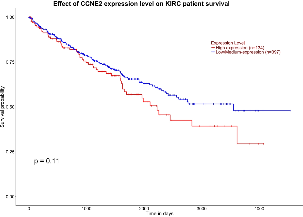

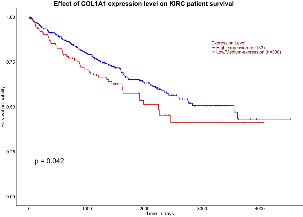

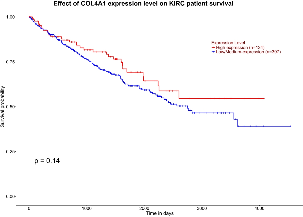

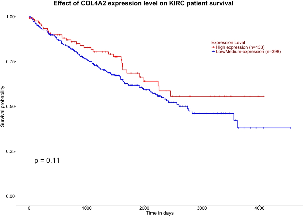

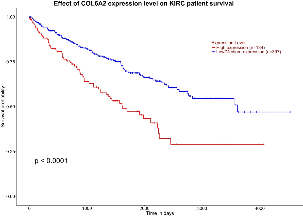

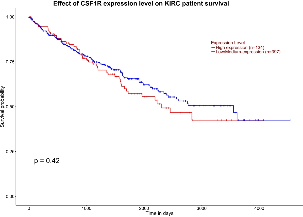

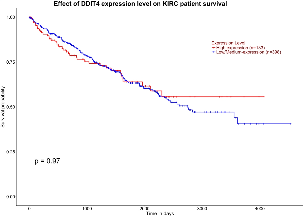

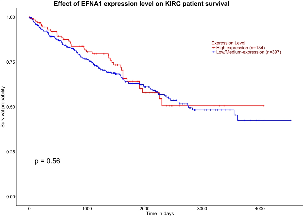

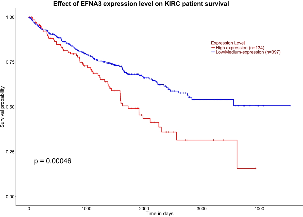

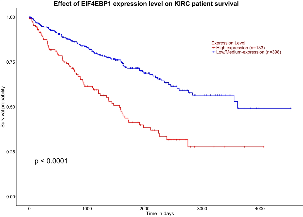

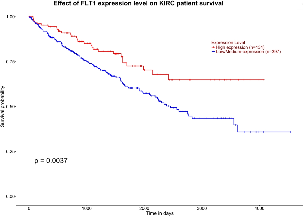

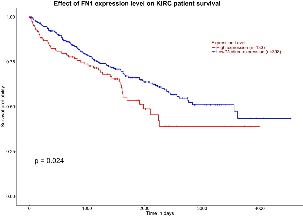

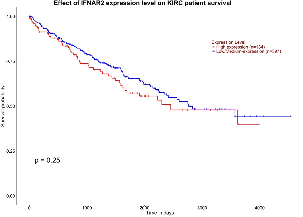

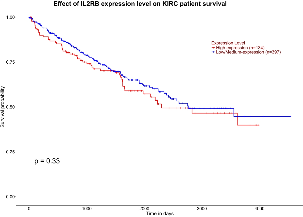

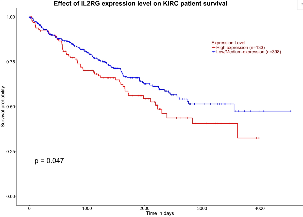

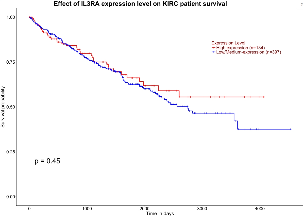

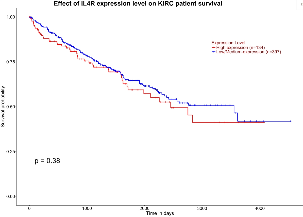

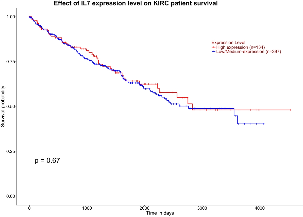

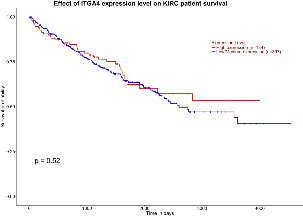

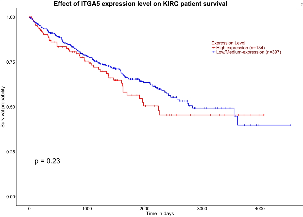

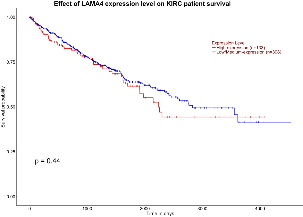

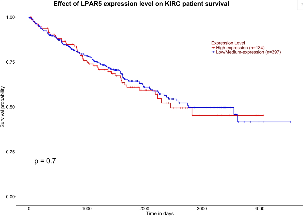

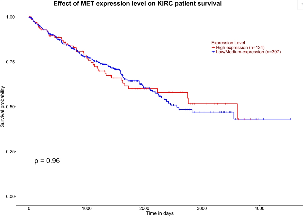

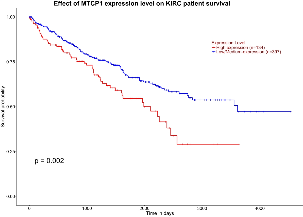

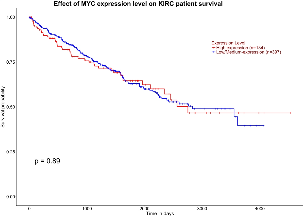

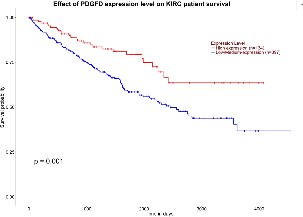

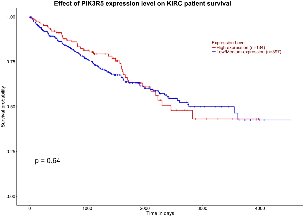

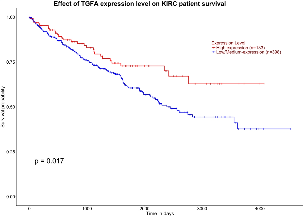

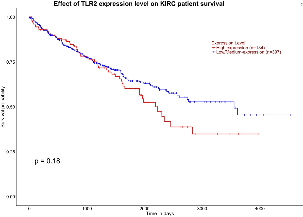

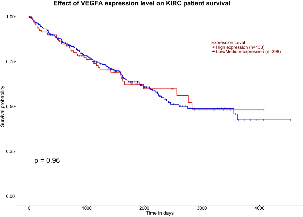

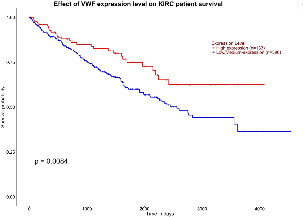


**Figure S2 The KM prognostic analysis of the 33 genes was downloaded based on the UALCAN website.**
